# Bridging the Gap: High‐Resolution *RHCE* Genotyping by Third‐Generation Sequencing Enhances Serological Interpretation of Weak Variants

**DOI:** 10.1155/humu/8527062

**Published:** 2026-08-21

**Authors:** Fan Wu, Li-Yan Sun, Shuang Liang

**Affiliations:** ^1^ Institution of Transfusion Medicine, Shenzhen Blood Center, Shenzhen, Guangdong, China

**Keywords:** *RHCE* gene, third-generation sequencing, weak RhCE variants

## Abstract

From January 2022 to June 2023, this study enrolled 124 healthy blood donors. Column agglutination and third‐generation sequencing (TGS) were used for *RHCE* genotyping and RhCE antigen typing. Additional serological screening was performed using conventional tube and column agglutination methods. Sanger sequencing was performed on 22 specimens with abnormal RhCE expression. Among the 124 samples, 102 exhibited normal RhCE expression whereas 22 showed abnormal expression; TGS‐predicted phenotypes disagreed with serological results in eight of the 102 normal‐expression samples and in seven of the 22 abnormal‐expression samples, with discrepancies attributed to reagent limitations and the presence of novel alleles. Indeed, six novel *RHCE* alleles were identified by TGS and their potential phenotypes inferred based on serological findings, demonstrating that conventional tube and column agglutination methods are effective in determining serological phenotypes, especially in cases of weak RhCE antigen expression, whereas TGS proves valuable for genotyping and elucidating the underlying causes of weakened expression.

## 1. Introduction

The RH blood group system is one of the most polymorphic systems of human red blood cells recognized by the International Society of Blood Transfusion (ISBT). In total, 56 antigens have been identified. The RH blood system is composed of two highly homologous genes, *RHD* and *RHCE*, which are closely linked and characterized by their opposing orientations. The *RHD* gene encodes the D antigen, whereas the *RHCE* gene encodes the corresponding C, c, E, and e antigens. The D, C, c, E, and e antigens are highly important in clinical blood transfusion, as the produced antibodies may lead to hemolytic disease of the fetus and newborn (HDFN) [1–3] and a hemolytic transfusion reaction [4–6].

Most studies have investigated the *RHD* gene and its expression using various sequencing approaches, including Sanger sequencing, next‐generation sequencing (NGS), and third‐generation sequencing (TGS) [7–9]; in contrast, the *RHCE* gene and its expression have received limited attention [10]. Like the *RHD* gene, the *RHCE* gene has many alleles produced by various mutations, such as genetic recombination, deletions, insertions, and missense mutations. More than 160 alleles of the *RHCE* gene have been reported, which may lead to weak, partial, or no expression of RhCE antigens [11–13].

Immunohematology laboratories regularly face several challenges or discrepancies between two serologic techniques. Thus, determining the serological phenotype via serological tests is difficult. Molecular biology techniques can be used to characterize the molecular background precisely, but there are several variants in the *RHCE* gene, making accurate determination of its phenotype through genotyping techniques difficult.

Major hospitals and blood centers have focused on *RHCE* genotyping and RhCE antigen typing to provide a matching basis for precise transfusion [14, 15]. However, subjective and objective factors such as different antibody reagents, different methodologies, and gene variants may affect the results of *RHCE* genotyping and RhCE antigen typing. The results of RhCE antigen typing vary when different monoclonal antibodies are used [16] and also depend on the method used, probably due to differences in the sensitivity [17]. Therefore, strategies need to be developed to obtain the RhCE serological phenotype accurately.

For *RHCE* genotyping, the following methods are used to study RhCE gene polymorphisms: polymerase chain reaction–sequence‐specific primer (PCR‐SSP) [18], Sanger sequencing [19], NGS [20], and high‐resolution dissolution curve [21]. PCR‐based *RHCE* genotyping methods have high false‐positive or false‐negative rates and cannot identify unknown variants. Sanger sequencing has a long read length and high accuracy, but its disadvantages are low sequencing throughput, slow speed, and high cost. Although NGS offers greater sequencing throughput than Sanger sequencing, its read length is too short to distinguish haploids, requiring greater reliance on population frequency data for predictions. In contrast, TGS features longer average read lengths, faster sequencing speeds, unbiased sequencing processes, and high accuracy. Therefore, it can resolve the challenge of distinguishing between haploid sequences and polygenic recombination events.

In this study, serological experiments and TGS were performed to type RhCE antigens and the *RHCE* gene, respectively. This approach provides a basis for accurately assessing the *RHCE* type of a sample and elucidates the reasons for the weak expression of RhCE antigens in some samples.

## 2. Materials and Methods

### 2.1. Serological Experiments With RhCE Antigens

From January 2022 to June 2023, EDTA‐anticoagulated blood samples from 124 healthy blood donors were collected, and serological screening tests of RhCE antigens were performed using an Automated Blood Grouping Analyzer 400 (Aikang MedTech Co. Ltd., China). The samples with agglutination strengths < 4+ were selected for confirmation.

The samples with weak expression in the screening tests were further typed for C, c, E, and e antigens using three antibody sets via the conventional tube technique (Set 1: anti‐C, clone MS‐24; anti‐c, clone MS‐33; anti‐E, clone MS‐80/258; anti‐e, clone MS‐16/21/63; Shanghai Blood Biomedicine Co. Ltd., China; Set 2: anti‐C, clone P3 25513G8; anti‐c, clone 951; anti‐e, clone P3GD512/MS63; anti‐E, clone 906; DIAGAST, Loos, France; Set 3: anti‐C, clone MS‐273; anti‐c, clone MS‐35; anti‐e, clone MS‐62/69; anti‐E, clone MS‐260/12; Changchun Brother BIO‐TECH Co. Ltd., China).

Moreover, two other Rh blood group antigen test cards were used for RhCE antigen typing (Bioxun Biotech Co. Ltd., China; Aikang Diagnostics Co. Ltd., China). The agglutination strengths of the samples < 3+, determined by the conventional tube technique, and < 4+, determined by the column agglutination technique, were regarded as weak expressions.

### 2.2. TGS for the *RHCE* Gene

#### 2.2.1. DNA Extraction From Samples

From January 2022 to June 2023, genomic DNA was extracted from 124 samples (Promega Biotech Co. Ltd., United States) using a Maxwell Clinical Sample Concentrator. The purity and concentration of the genomic DNA were evaluated via spectrophotometry (NanoDrop 1000 Spectrophotometer, Wilmington, Delaware, United States).

#### 2.2.2. Long‐Range PCR (LR‐PCR) Amplification for Single‐Molecule Real‐Time (SMRT) Sequencing

Eight primer pairs were developed to amplify the *RHCE* gene, ensuring a 1‐kb overlap between each amplification segment (Figure S1). LR‐PCR was performed using KOD FX Neo (TOYOBO BIOTECH Co. Ltd., Japan), and the PCR cycling conditions were set as follows: KOD Neo FX Buffer, 10.5 *μ*L; dNTPs, 4 *μ*L; primers, 9 *μ*L; gDNA, 1 *μ*L; and KOD Neo FX, 0.5 *μ*L. The PCR products were verified through agarose gel electrophoresis and purified using 0.6x AMPure PB beads (Pacific Biosciences).

#### 2.2.3. Library Preparation and SMRT Sequencing

SMRT libraries were constructed using a one‐step method that combines DNA damage repair, end repair, and adapter ligation to generate uniquely barcoded presequencing libraries. The reaction mixture was prepared in a total volume of 15 *μ*L, comprising 1.5 *μ*L of 10x T4 DNA ligase buffer, 0.1 *μ*L of 10 mM dNTPs (each), 0.1 *μ*L of 100 mM ATP, 0.3 *μ*L of T4 DNA polymerase, 0.3 *μ*L of T4 polynucleotide kinase, 0.45 *μ*L of T4 DNA ligase, 4 *μ*L of PCR product, 2 *μ*L of barcoded adapter, and 6.25 *μ*L of nuclease‐free ddH_2_O.

To prepare the presequencing libraries, 120–250 ng of purified PCR product was mixed with the enzyme mixture and incubated at various temperatures for different durations. Exonuclease I and exonuclease III were added to eliminate failed ligation products, and the final prelibrary was purified via 0.6x AMPure PB beads. For sequencing multiple samples, the prelibraries were combined based on equal masses and further purified twice using 0.45x AMPure PB beads.

To complete the library, sequencing enzymes and primers were bound using Sequel Binding Kit 2.2 and Internal Control Kit 1.0 (Pacific Biosciences of California Inc., United States). Next, 150‐pM DNA‐polymerase complexes were loaded onto the Sequel II platform, and the library was sequenced for 20 h.

#### 2.2.4. Data Analysis and DNA Variant Calling

SMRT Link v10.1.0 software (Pacific Biosciences of California Inc., United States) was used for the primary analysis of the output data. Initially, the raw reads underwent demultiplexing, and the barcode sequences were automatically detected after the runs were completed. Next, the subreads were analyzed using the CCS 12.3.0 software to generate circular consensus sequences (CCS), which were subsequently aligned with the GRCh38 human reference genome by the pbmm2 aligner. The targeted *RHCE* CCS fragments were processed in batches, and the corresponding CCS reads were remapped to the reference genome (ISBT *RHCE* gene) by pbmm2. To detect single‐nucleotide variants (SNVs) and minor indels, variant analysis was performed using DeepVariant v1.2.0. The allele designation followed the recommendations of the ISBT working group. Major structural variations, including exon translocation, were identified using an in‐house software module through the assembly of the *RHCE*–*RHD* region.

### 2.3. Sanger Sequencing for *RHCE* Gene and *RHD* Gene

Sanger sequencing was performed on the 22 samples that showed weak serological expression. The PCR primers and amplification/sequencing procedures were based on those previously established by our research team [22]. The sequencing results were aligned and analyzed against the *RHCE* gene reference sequence BN000065 from GenBank.

To conduct a comprehensive analysis of the six samples carrying *RHCE* ∗ *CE* and *RHCE* ∗ *cE* alleles, we performed Sanger sequencing of all 10 exons of the *RHD* gene and their flanking intronic regions. The primers and protocols for PCR amplification and sequencing were adopted from our previous study [23]. The resulting sequences were aligned and compared against the *RHD* reference sequence available in the GenBank database (accession number: BN000065).

## 3. Results

### 3.1. Serological Screening Results of the RhCE Antigen

Among the 124 samples analyzed, 102 presented normal RhCE antigen expression according to the serological results, 18 presented variable weak expression (w+~3+), three presented negative expression, and one presented mixed agglutination of RhCE antigens.

### 3.2. Results of TGS

#### 3.2.1. TGS Analysis of 102 Samples With Normal RhCE Antigen Expression

Among the 102 samples analyzed, eight exhibited discordance between the serological phenotypes and TGS predictions (Table 1). Three of these were predicted to have weak e antigen expression by TGS (Table 2), whereas serological tests showed normal reactivity. Further analysis revealed that two of these three samples carried a combination of *RHCE* ∗ *Ce* and *RHCE* ∗ *ce.01* alleles, whereas the third sample presented a hybrid genotype of *RHCE* ∗ *ce* and *RHCE* ∗ *ce.01*. The variations in the e antigen of *RHCE* ∗ *ce.01* allele leads to the weak expression of the e antigen detectable only by monoclonal anti‐e antibodies. However, we used polyclonal anti‐e reagents, which explains why the expression of the weakened e antigen predicted by TGS was not detected via serological testing.

**Table 1:** Results of serological screening tests and third‐generation sequencing among 102 specimens.

Rh serology	The number of serological phenotypes consistent with the predicted phenotypes from the third‐generation sequencing results	The number of predicted phenotypic inconsistencies between serological phenotypes and third‐generation sequencing results
CCee	53	1
CcEe	22	1
Ccee	11	4
ccee	2	1
ccEe	4	0
ccEE	2	0
CCEe	0	1

*Note:* Specimens whose serologic phenotypes are inconsistent with the predicted phenotypes from the third‐generation sequencing results include specimens whose phenotypes cannot be accurately predicted from the third‐generation sequencing results (e.g., the presence of new alleles cannot predict their serologic phenotypes).

**Table 2:** The information about eight samples whose serologic phenotypes were not completely consistent with the phenotypes predicted by third‐generation sequencing.

Code of specimen	Serological phenotype	RHCE Allele 1	RHCE Allele 2	Predicted phenotype	GenBank Accession Number
1	Ccee	RHCE∗Ce	RHCE∗ce.01	Ccee^weak^	
2	Ccee	RHCE∗Ce	RHCE∗ce.01	Ccee^weak^	
3	ccee	RHCE∗ce	RHCE∗ce.01	ccee^weak^	
4	CCee	RHCE∗Ce	RHCE∗Ce.27	CC^weak^ee^weak^	
5	CcEe	RHCE∗cE	c.361A>T in RHCE∗Ce	CcE^weak^e	
6	Ccee	RHCE∗ce	c.105C>T in RHCE∗Ce	Ccee	PQ570501
7	CCee	RHCE∗Ce	c.806A>G in RHCE∗Ce	CCee	PQ570500
8	CCEe	RHCE∗Ce	c.48G>T in RHCE∗CE	CCEe	PQ570502

*Note:* Three novel alleles we found have been attached to the GenBank accession numbers.

The TGS data revealed that Specimen 4 carried *RHCE* ∗ *Ce*/*RHCE* ∗ *Ce.27* alleles. Previous studies have speculated that the *RHCE* ∗ *Ce.27* allele might decrease the expression of C and e antigens. Based on this evidence, this sample was inferred to have weak C and weak e antigen expression. However, the serological phenotype of the sample was identified as CCee, demonstrating the weakened C/e antigen expression predicted by TGS. The mechanism underlying this discrepancy needs to be elucidated.

Moreover, four specimens contained alleles with unreported serological phenotypes (Codes 5, 6, 7, and 8), preventing accurate phenotypic prediction through TGS analysis (Table 2). For Sample 5, one allele was *RHCE* ∗ *cE*, and the other allele was c.361A>T in *RHCE* ∗ *Ce* (GenBank: LN714470.1), which can lead to the conversion of methionine to leucine at position 121. However, the corresponding serological results of this allele have not been reported, although a similar variant in a different allele context has been previously associated with weak E antigen expression [4]. The serological results of this study showed that the serotype of the specimen with c.361A>T in the *RHCE* ∗ *Ce* allele resulted in the CcEe phenotype, with no evidence of a decrease in the expression of the E antigen. These findings indicated that the c.361A>T *RHCE* ∗ *Ce* allele may not decrease the expression of the E antigen.

For Specimen 6, one allele was identified as *RHCE* ∗ *ce*, and the other represented a novel allele (c.105C>T in the *RHCE* ∗ *Ce* allele). A similar silent mutation has been previously documented in the *RHCE* ∗ *ce* allele without affecting antigen expression [22], which matched our observation of normal Ccee serology without a decrease in antigen expression. These findings indicated that c.105C>T in the *RHCE* ∗ *Ce* allele may not affect the expression of the RhCE antigen.

We found three novel alleles among 102 samples via TGS. Along with the novel allele c.105C>T in the *RHCE* ∗ *Ce* allele described above, we identified two additional novel alleles: c.806A>G in *RHCE* ∗ *Ce* and c.48G>T in the *RHCE* ∗ *CE* alleles. Among them, c.806A>G in *RHCE* ∗ *Ce* was the novel allele of Specimen 7, and the other allele of Specimen 7 was *RHCE* ∗ *Ce*. The c.806A>G variant may result in the substitution of tryptophan with cysteine at position 269 (p.Trp269Cys). As this mutation has not been reported, its phenotypic effect cannot be predicted from sequencing alone. However, the CCee phenotype observed with normal antigen expression suggests that this novel variant does not impair RhCE antigen detection via routine serological tests. Similarly, the novel allele c.48G>T in *RHCE* ∗ *CE* of Specimen 8 has not been reported. This variant is functionally analogous to the known c.48G>C mutation, as both substitutions replace tryptophan with cysteine at position 16 (p.Trp16Cys). Therefore, we concluded that the c.48G>T variant in the *RHCE* ∗ *CE* allele does not significantly alter the expression of the C antigen, as indicated by the concordance between the TGS predictions and the observed CCee serological phenotype in Specimen 8.

#### 3.2.2. TGS Analysis of Specimens With Weak RhCE Antigen Expression

The serological findings for 18 specimens exhibiting weak RhCE antigen expression were presented in Table 3.

**Table 3:** Detailed information of 22 samples with weak expression of RhCE antigens.

The total number of samples	Rh serology	RHCE Allele 1	RHCE Allele 2	Predicted phenotype	GenBank Accession Number
C^weak^					
2	C^weak^cEe	RHCE∗ce.39	RHCE∗cE	C^weak^cEe	
2	C^weak^cee	RHCE∗ce.39	RHCE∗ce	C^weak^cee	
1	C^weak^C^weak^ee	RHCE∗ce.39	RHCE∗ce.39	C^weak^C^weak^ee	
1	C^weak^cEe	RHCE∗Ce.04	RHCE∗cE	C^weak^cEe	
1	C^weak^cEE	c.208C>T in RHCE∗CE	RHCE∗cE	C^weak^cE^weak^E^weak^	PQ497785
6	C^weak^cEE	RHCE∗CE	RHCE∗cE	C^weak^cEE	
E^weak^					
2	CCE^weak^e	RHCE∗Ce	c.281T>C, c.282G>T in RHCE∗cE	CcEe	
c^weak^E^weak^					
1	Cc^weak^E^weak^e	RHCE∗Ce	c.635‐8insG in RHCE∗cE	CcEe	OP455108
2	Cc^weak^E^weak^e	RHCE∗Ce	c.512 A>C in RHCE∗cE	CcEe	OP501867
Others					
1	CcEe mf	RHCE∗Ce	RHCE∗cE	CcEe	
1	D‐‐	RHCE∗CeN.07	RHCE∗CeN.08	Null	
1	D‐‐	RHCE∗NEW	RHCE∗CeN.07	Null	
1	D‐‐	RHCE∗NEW	RHCE∗CeN.08	Null	

*Note:* Three novel alleles we found have been attached to the GenBank accession numbers.

Abbreviation: mf, representative of mixed field agglutination.

##### 3.2.2.1. TGS Analysis of Specimens With Weak C Antigen Expression

Among these 18 samples, 13 samples presented weak C antigen expression, and the serological phenotype of 12 samples aligned with the phenotype predicted by the TGS, with only one sample exhibiting a genotype–phenotype discordance. Five of these 12 concordant specimens carried the *RHCE* ∗ *ce.39* (C.308C>T) allele, which can silence c antigen expression and reduce C antigen strength [14]. The companion alleles identified in these five cases were presented in Table 3. Additionally, one specimen was found to carry the *RHCE* ∗ *Ce.04* allele, which was associated with weakened or absent C antigen expression [24]. In combination with its *RHCE* ∗ *cE* allele, TGS predicted a C^weak^cEe phenotype that matched the observed serological results. Six specimens exhibited the C^weak^cEE phenotype (detailed in Table 3), and these individuals carried the *RHCE* ∗ *CE* and *RHCE* ∗ *cE* alleles along with *RHD* ∗ *01.* There is evidence suggesting that the observed C^weak^ phenotype was associated with the DCE haplotype.

The specimen with a serological phenotype that was inconsistent with the phenotype predicted by TGS presented a C^weak^cEE serological phenotype, whereas TGS analysis revealed *RHCE* ∗ *cE* and *RHCE* ∗ *CE* (c.208C>T) alleles. The c.208C>T variant results in an arginine‐to‐tryptophan substitution at position 70 (p.Arg70Trp). Although some researchers have speculated that this variant in the *RHCE* ∗ *cE* allele (*RHCE* ∗ *cE.22*) may reduce the expression of the E antigen, serological evidence remains unavailable. In this study, TGS predicted the C^weak^cE^weak^E^weak^ phenotype, which conflicts with the observed serological results. This finding suggests that c.208C>T primarily affects C antigen expression when it is present in the *RHCE* ∗ *CE* allele rather than the *RHCE* ∗ *cE* allele.

##### 3.2.2.2. TGS Analysis of Specimens With Weak E Antigen Expression

Among 18 specimens with weakened RhCE antigen expression, two specimens exhibited attenuated E antigen reactivity. Both samples shared the *RHCE* ∗ *cE* allele variants c.281T>C and c.282G>T (p.Leu94Pro, GenBank accession: MN725054). Although these variants have been genetically documented, their serological consequences remain unreported. Therefore, its phenotype cannot be predicted by TGS. In this study, the serological phenotype of these two samples was identified as CCE^weak^e, suggesting that the p.Leu94Pro substitution may affect the expression of the c and E antigens.

##### 3.2.2.3. TGS Analysis of Specimens With Weak c and E Antigen Expression

Three of the 18 samples presented weak expression of c and E antigens. The genotypes of two samples were *RHCE* ∗ *Ce* and c.512A>C for the *RHCE* ∗ *cE* alleles. The mutation c.512A>C changes the original histidine to proline (p.His171Pro), leading us to hypothesize that it might influence the expression of c and E antigens. Therefore, the serological phenotype of c.512A>C in the *RHCE* ∗ *cE* allele might be classified as c^weak^E^weak^. The other sample has a single‐base mutation in the intron region, with one allele *RHCE* ∗ *Ce* and a novel allele c.635‐8insG in *RHCE* ∗ *cE*, suggesting that the serological phenotype of the novel allele c.635‐8insG in *RHCE* ∗ *cE* may be c^weak^E^weak^.

#### 3.2.3. TGS Analysis of Specimens With the D‐‐ Phenotype

Serological typing identified three specimens exhibiting the D‐‐ phenotype. Sanger sequencing failed to identify their underlying genetic basis, whereas TGS successfully resolved complex structural variations. TGS identified one sample carrying both *RHCE* ∗ *CeN.07* and *RHCE* ∗ *CeN.08* alleles, which were documented null variants resulting in the complete absence of the expression of C and e antigens. Compared with traditional Sanger sequencing, TGS enables the precise determination of recombination breakpoints for the *RHCE* ∗ *CeN.07* and *RHCE* ∗ *CeN.08* alleles, as summarized in Table S1. Specifically, the recombination start and end intervals for *RHCE* ∗ *CeN.07* are located at 149−997 to 147−939 and 1227+2545 to 1227+2831, respectively; for *RHCE* ∗ *CeN.08*, the start interval spans 149−997 to 149−939, and the end interval spans 1074−327 to 1153+890.

In the remaining two samples, TGS revealed that one allele in each sample was *RHCE* ∗ *CeN.07* or *RHCE* ∗ *CeN.08*, whereas the other allele of these two samples was suspected to have undergone recombination of large fragments. Owing to the inherent limitations of the amplicon‐based methodology used in TGS, we were able to determine only the recombination start sites for the two recombinant alleles, whereas the corresponding end sites could not be identified (Figure S2). The recombination fragments of these two alleles were different, and the unique recombination patterns observed in these alleles (Figure S2) suggested that they may represent previously undocumented alleles. The TGS method used in this study could not resolve the complete sequences of these two novel alleles. The observed D‐‐ phenotype in both samples suggested that these recombinant variants probably abrogate all RhCE antigen expression.

#### 3.2.4. TGS Analysis of Samples With Mixed Agglutination of RhCE Antigens

One specimen showed mixed agglutination for the C, c, E, and e antigens despite genotyping as *RHCE* ∗ *CcEe* by TGS. However, we found that the read count for one haploid was about half that of the other by TGS, leading us to speculate about the potential existence of a third haploid. Thus, the sample phenotype cannot be reliably determined through TGS data alone.

### 3.3. Serological Characterization of Novel Alleles in Weak RhCE Expressions

The serological analysis confirmed weak RhCE antigen expression in three specimens harboring novel alleles (Table 4). Specimen 1 showed notable serological discrepancies in E antigen detection across different methodologies and monoclonal antibodies. Column agglutination revealed strong reactivity (3+) with anti‐E (clone MS‐260/12) but negative reactivity with anti‐E (clone MS‐258/MS906). When the conventional tube technique was used, we found differential E antigen reactivity: negative results with anti‐E (clone MS‐80/258) and weak reactions with anti‐E (clone MS‐260/12 or 906). Our findings showed that the serological results for a given antigen can vary significantly depending on the monoclonal antibody clone used, even when identical testing methodologies were implemented.

**Table 4:** Serological confirmation results of three samples with new alleles.

Source of reagents sample name		Sample 1	Sample 2	Sample 3
Rh serology		Cc^weak^E^weak^e	Cc^weak^E^weak^e	C^weak^cEE
Rh genotype		c.635‐insG in RHCE∗cE, RHCE∗Ce	c.512A>C in RHCE∗cE, RHCE∗Ce	c.208C>T in RHCE∗CE, RHCE∗cE
Shenzhen Aikang Reagent Co. Ltd. (column agglutination)	C(MS‐24)	4+	4+	3+s
	c(MS‐33)	3+	4+	4+
	E(MS‐260/12)	3+	2+	4+
	e(MS‐21/16/63)	4+	4+	—
Changchun Boxun Biotechnology Co. Ltd. (column agglutination)	C(MS‐24)	4+	4+	3+
	c(MS‐33)	±	4+	4+
	E(MS‐258/MS906)	—	3+s	4+
	e(MS‐16/21/63)	4+	4+	—
Shanghai Blood Biomedicine Co. Ltd. (conventional tube)	C(MS‐24)	4+	3+	1+
	c(MS‐33)	w+	2+s	3+s
	E(MS‐80/258)	—	2+s	3+s
	e(MS‐16/21/63)	4+	3+	—
Changchun Bode Biotechnology Co. Ltd (conventional tube)	C(MS‐273)	3+	3+	1+
	c(MS‐35)	1+	3+	3+s
	E(MS‐260/12)	1+w	w+	4+
	e(MS‐62/69)	3+	3+	—
DIAGAST (conventional tube)	C(P3X25513G8+MS‐24)	4+	3+s	3+
	c(951)	1+	3+	3+
	E(906)	1+	—	3+
	e(P3GD512+MS63)	4+	3+	—

*Note:* “w” indicates weak positive agglutination; “s” denotes strong agglutination.

We also found that antigen detection results vary significantly across testing methodologies, even when identical antibody clones are used (Table S2). Specimen 2 demonstrated differential c antigen reactivity when tested with three MS‐33 clone anti‐c reagents: strong reactivity (4+), determined by column agglutination testing, and weak reactivity (2+s), determined by the conventional tube technique. Similarly, Specimen 3 demonstrated method‐dependent variation in C antigen detection when tested with MS‐24 clone anti‐C reagents: consistent strong reactivity (3+/3+s) across two detection systems, determined by column agglutination testing, and weak reactivity (1+), determined by the conventional tube technique.

## 4. Discussion

The accurate classification of Rh variants is crucial for transfusion safety, as their association with partial antigen expression increases alloimmunization risk, whereas their lack of conserved epitopes limits donor compatibility. Molecular genotyping can accurately identify the true blood group genotype. TGS, including the ABO blood group system [25] and the MNS blood group system [26], has been extensively used in recent years for blood group genotyping. Recent studies have used TGS for comprehensively characterizing the *RHD* and *RHCE* genes, demonstrating its reliability for RH blood group system genotyping [27]. However, previous studies primarily analyzed specimens with normal RhCE antigen expression, most of which carry only SNVs detectable by conventional methods, including Sanger sequencing, NGS, and TGS. In these previous studies, TGS was applied mainly to identify novel alleles and predict their potential phenotype in combination with serological findings. However, these applications fail to fully demonstrate the unique advantages of TGS for analyzing the RH blood group system.

Our research group speculated that TGS is more relevant for studying specimens with weak RhCE antigen expression, as these cases are significantly more likely to harbor complex genetic variations than normal expressors. It is the initial application of TGS along with Sanger sequencing for samples exhibiting weak RhCE antigen expression. To validate the accuracy of TGS for *RHCE* genotyping, we performed a comparative analysis using both TGS and Sanger sequencing on the 22 samples with weak RhCE antigen expression. TGS successfully identified the underlying genotypes in all cases, particularly excelling in detecting novel alleles and large structural variations (Figure S3). In contrast, due to its limited read length and inability to perform haplotype phasing, Sanger sequencing failed to detect complex structural variations in four cases (including D– and mixed agglutination specimens). Furthermore, although Sanger sequencing could identify novel mutation sites, it could not determine the complete sequences of six novel alleles. This comparative analysis confirmed the superior resolution of TGS for characterizing weak RhCE variants, particularly for structural alterations beyond the detection limits of Sanger sequencing.

This study found that although Sanger sequencing can identify specimens containing alleles with multiple mutations, it cannot be used to determine the specific haploids of these mutations (e.g., the novel alleles presented in Table 3). Such cases may require cloning sequencing for verification. In contrast, TGS provides direct haplotype resolution, which is important for accurately interpreting weak RhCE variants with multiple polymorphisms. TGS analysis of six samples with weak C antigen expression revealed the presence of *RHCE* ∗ *CE* and *RHCE* ∗ *cE* alleles. Subsequent *RHD* genotyping showed that all six samples carried the *RHD* ∗ *01* alleles, which were consistent with the R_Z_ haplotype and closely related to the expression of C^weak^. This finding indicated that TGS is a necessary method for detecting weak Rh variants with suspected haplotype associations. Additionally, some specimens (e.g., those with the D–– phenotype in Table 3) exhibited recombination or deletion of large fragments that could not be resolved by Sanger sequencing owing to its limited read length and inability to span complex structural variations, whereas TGS deciphered these alterations through its long‐read capability. Through comprehensive analysis of 22 samples with weak RhCE antigen expression, TGS identified three novel *RHCE* alleles, large fragment recombinations in two samples, large fragment deletions in one sample (harboring *RHCE* ∗ *CeN.07* and *RHCE* ∗ *CeN.08* null alleles), and a possible third haploid in one sample, all of which were undetectable by Sanger sequencing. This finding showed that TGS is better at characterizing weak RhCE antigen variants, as it can efficiently and precisely identify complex gene variants and elucidate the causes underlying the decrease in the expression of RhCE antigens that conventional methods cannot resolve.

Besides using TGS to identify specimens exhibiting weak expression, TGS was also used to analyze 102 specimens with normal expression of the RhCE antigen. Among these 102 specimens, the phenotypes predicted by TGS for eight specimens were inconsistent with the serological phenotypes. In three of these eight specimens (Table 2, Specimens 1–3), this discrepancy was attributed to limitations in the ability of polyclonal anti‐e antibodies to detect weak expression of the e antigen. However, previous studies have reported that monoclonal anti‐e antibodies (e.g., clones MS‐16, MS‐17, MS‐19, and MS‐69) can detect these weak e variants. This highlights the complementary value of TGS in resolving such discordances through comprehensive genotypic analysis. The remaining four cases involved novel alleles or variants with uncharacterized serologic phenotypes (Table 2, Specimens 5–8). TGS not only provides definitive genotypic characterization of these variants but also has an advantage in identifying previously undocumented alleles. In one specimen, the discordance between the TGS‐predicted phenotype and the serological result reported in relevant documents remained unresolved (Table 2, Specimen 4). Whether this discrepancy occurred due to the limitations of current serological reagents (e.g., antibody clone specificity) or regulatory mechanisms affecting antigen expression requires further investigation. To summarize, combining genotypic data from sequencing with serological phenotyping is necessary to achieve accurate RhCE antigen characterization, particularly for specimens exhibiting ambiguous or weak expression patterns.

Several traditional and advanced techniques have been used for identifying blood group system antigens [28]. The conventional tube technique and column agglutination technology are the most widely used methods in clinical and research settings. This study demonstrated that antibody clonal selection and testing methodology significantly affect RhCE antigen typing results, which agrees with the findings of other studies. As shown in Table 4, weak E or C expression was not detected in certain antibody clones, potentially leading to the misclassification of phenotypes. When this occurs in blood donors, such antigenic mismatches may stimulate transfusion recipients to develop alloantibodies [29]. Besides analyzing the primary study specimens, serological experiments were also conducted on some specimens with weak expression (Table S1). Our findings revealed that even when identical methodologies and antibody clones are used, the reaction intensities may vary. This variability may be associated with antibody manufacturing processes, titers, or other reagent‐specific factors. These observations highlighted the necessity of comprehensive RhCE phenotyping, which combines Rh blood group antigen detection cards with multiple monoclonal antibody clones to minimize phenotype misinterpretations, which is especially critical for specimens exhibiting weak antigen expression or harboring novel alleles.

Although TGS has a higher upfront cost than conventional serology and Sanger sequencing, its cost‐effectiveness must be judged by the clinical problems it addresses. For normal blood donor screening, serological methods remain the most cost‐effective choice. However, for samples with weak or discrepant RhCE expression, the costs of unresolved typing—including repeat testing and clone sequencing—far outweigh the per‐sample cost of TGS. In these difficult cases, TGS provides a complete genotypic solution in a single run, identifying novel alleles, haplotypes, and structural variants. Thus, we advocate a targeted TGS strategy for specimens with ambiguous or weak serological results, which balances diagnostic accuracy and resource use effectively.

## 5. Conclusion

To summarize, the clinical determination of *RHCE* typing requires a multimodal approach. First, initial serological screening needs to be conducted to identify specimens with weak antigen expression, which must then undergo confirmatory testing using at least two antibody clones with different epitope specificities and complementary techniques (e.g., the tube technique and column agglutination) to decrease the variability that can be caused by the reagents or the testing methods. Next, TGS should be performed on specimens with weak antigen expression, as it can solve genetic complexities that traditional methods cannot detect, such as novel alleles, hybrid genes, and large structural variants. By combining the genotypes obtained via TGS analysis with more accurate serological phenotyping, clinicians can achieve several critical objectives. First, they can determine unclear antigen expression patterns. Second, they can identify the molecular mechanisms underlying a decrease in antigen expression, such as promoter variants or recombination events. Third, they can increase the compatibility between donors and recipients to decrease the risk of alloimmunization. This combined strategy establishes TGS as an essential tool in modern transfusion medicine, particularly for resolving the increasing variety of weak RhCE variants with a level of precision that serology alone cannot achieve.

## Author Contributions

Fan Wu and Li‐Yan Sun conceived and designed the study; Fan Wu and Li‐Yan Sun performed the experiments and analyzed the data; Shuang Liang contributed reagents/materials/analysis tools; Li‐Yan Sun and Fan Wu wrote the manuscript; Shuang Liang critically revised the work. Fan Wu and Li‐Yan Sun contributed equally to this work.

## Funding

This project was supported by the Sanming Project of Medicine in Shenzhen Municipality (SZSM202311032), Natural Science Foundation of Shenzhen Municipality (JCYJ20230807154000002), Guangdong Medical Research Foundation (B2025582 and A2024098), and Shenzhen Key Discipline Project of Blood Transfusion Medicine (SZXK070).

## Disclosure

All authors reviewed and approved the final version of this manuscript.

## Ethics Statement

All participants signed the informed consent form, and the study was approved by the Shenzhen Ethics Committee (No.: SZBCMEC‐2022‐024).

## Conflicts of Interest

The authors declare no conflicts of interest.

## Supporting information


**Supporting Information 1** Additional supporting information can be found online in the Supporting Information section. Table S1: Recombination region haplotypes of *RHCECeN.07* and *RHCECeN.08*. Table S2: Serological confirmation results of some specimens with weak expression. Figure S1: Full‐length amplification and sequencing of the *RHCE* gene using third‐generation sequencing technology. Figure S2: Haplotype maps of two D‐‐ genes specimens carrying novel recombinant *RHCE* alleles. Figure S3: IGV views of structural variants identified in four specimens.

## Data Availability

The data supporting the conclusions of this article are included within the article (and its additional files).
