# Supplementary material for: Bridging the Gap: High‐Resolution RHCE Genotyping by Third‐Generation Sequencing Enhances Serological Interpretation of Weak Variants
Source: Hum Mutat. 2026 Aug 21;2026:8527062. doi: 10.1155/humu/8527062 (PMC13494661; doi:10.1155/humu/8527062)
Supplement: Supplementary file 1 [file HUMU-2026-8527062-s001.pdf]

Table S1 Recombination region haplotypes of *RHCECeN.07* and *RHCECeN.08*

	149-997	149-939	1074-329	1074-327	1153+890	1153+898	1227+2545	1227+2831
RHD	G	C	G	G	A	C	G	T
RHCE-D(3-9)-CE	A	C	G	G	A	C	G	C
RHCE-D(3-8)-CE	A	C	G	G	C	A	A	C
RHCE*ce	A	T	A	A	C	A	A	C

Note: *RHCECeN.07*: RHCE-D(3-8)-CE; *RHCECeN.08*: RHCE-D(3-9)-CE.

Table S2: Serological confirmation results of some specimens with weak expression.

Source of reagents / Sample name		Sample IV	Sample V	Sample VI
Rh serology		CCE^{weak}e	C^{weak}cEe	CcEe mf
Rh genotype		c.281T>C, c.282G>T in RHCE*cE,RHCE*Ce	RHCE*ce.39, RHCE*cE	-
Shenzhen Aikang Reagent Co., LTD	C(MS-24)	4+	3+	3+mf
	c(MS-33)	-	4+	4+mf
	E(MS-260/12)	1+w	4+	4+mf
	e(MS-21/16/63)	4+	4+	3+mf
Changchun Boxun Biotechnology Co., LTD	C(MS-24)	3+s	-	3+mf
	c(MS-33)	-	4+	4+mf
	E(MS-258/MS906)	-	4+	4+mf
	e(MS-16/21/63)	3+s	4+	3+mf
Shanghai Blood Biomedicine Co., Ltd	C(MS-24)	3+	w+	1+mf
	c(MS-33)	-	3+	3+mf
	E(MS-80/258)	-	3+	3+mf
	e(MS-16/21/63)	3+	3+	1+mf
Changchun Bode Biotechnology Co., LTD	C(MS-273)	3+	±	1+mf
	c(MS-35)	3+	3+	3+mf
	E(MS-260/12)	w+	3+	3+mf
	e(MS-62/69)	3+	3+	1+mf

DIAGAST	C(P3X25513G8+MS-24)	4+	1+	1+mf
	c(951)	-	2+	3+mf
	E(906)	w+	3+	3+mf
	e(P3GD512+MS63)	3+	3+	1+mf

Note: "mf" stands for mixed field agglutination; "w" indicates weak positive agglutination; "s" denotes strong agglutination.

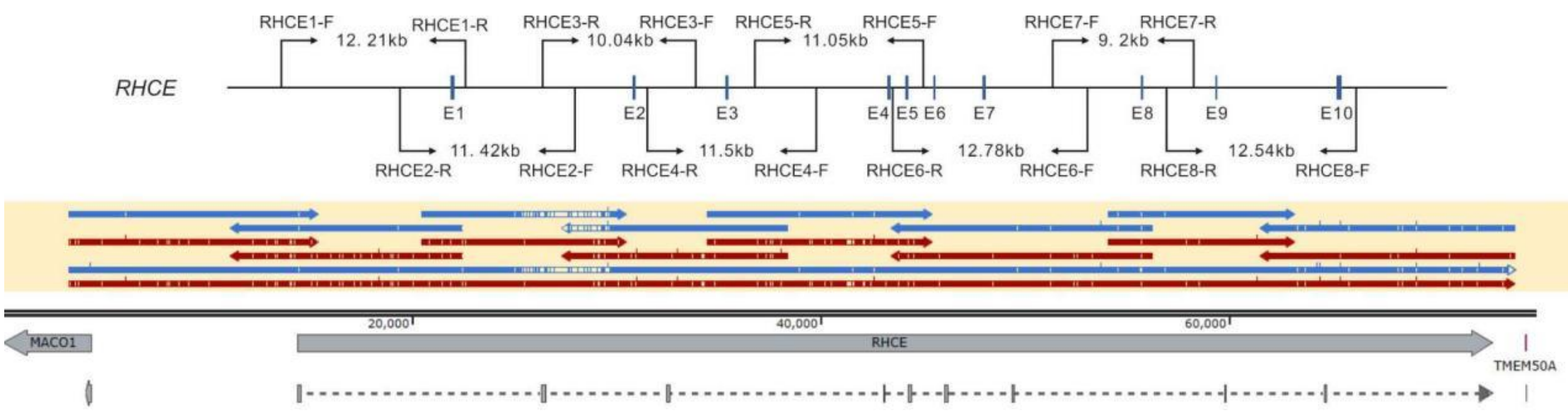

Figure S1: Full-length amplification and sequencing of the *RHCE* gene using third-generation sequencing technology.

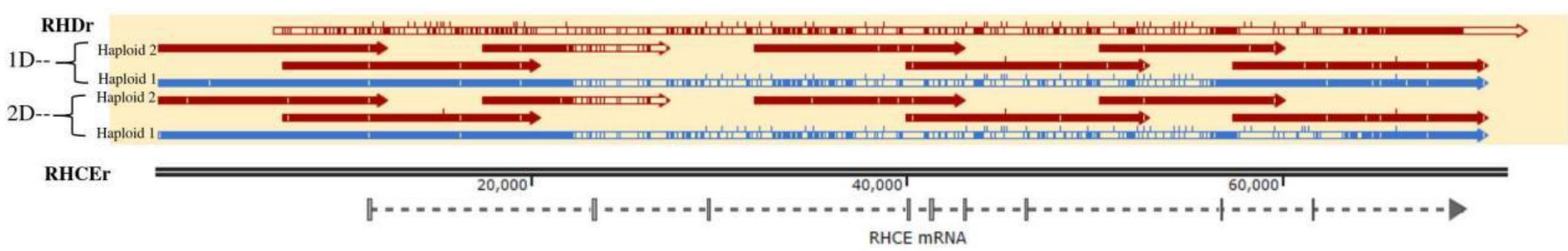

Figure S2: Haplotype maps of two D-- specimens carrying novel recombinant *RHCE* alleles.

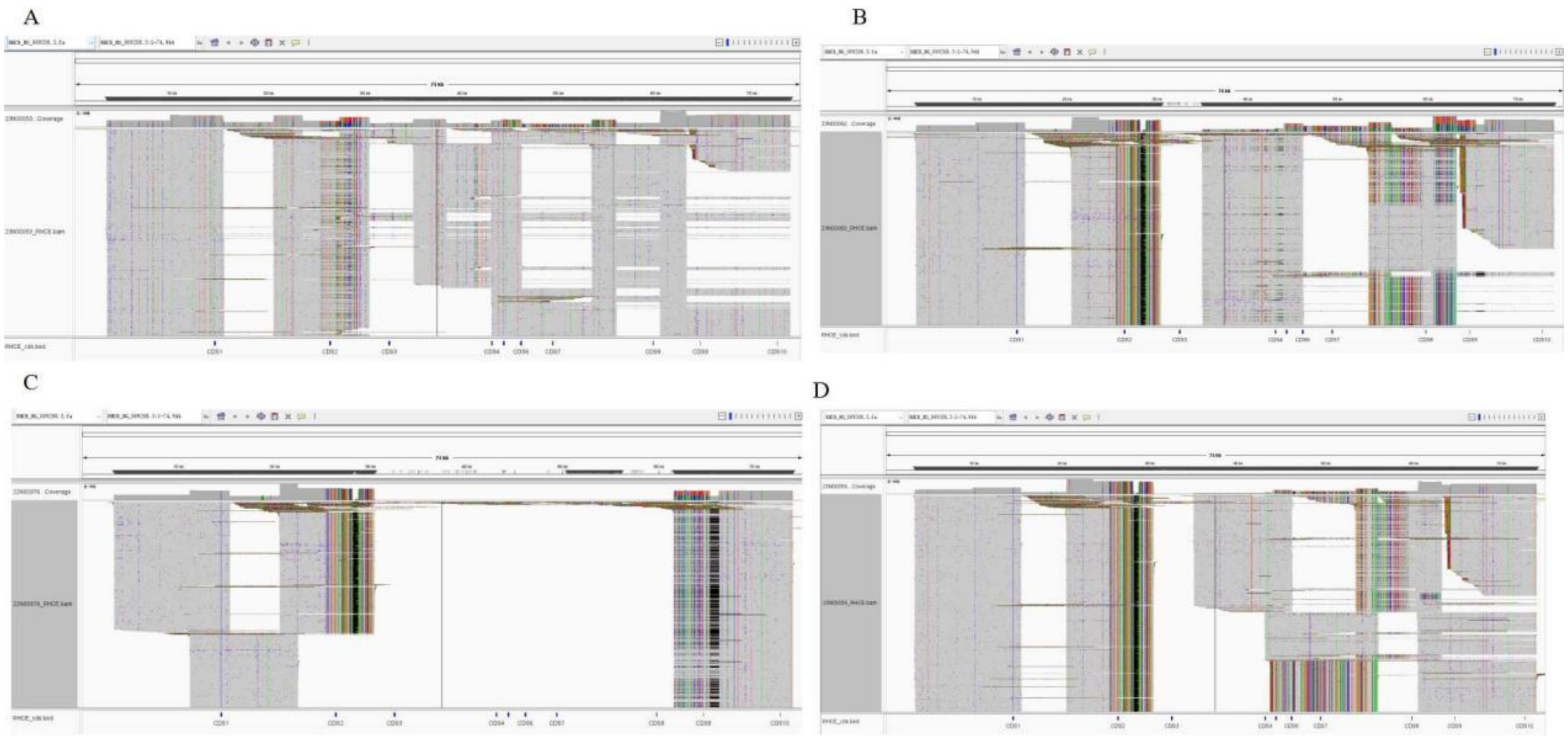

Figure S3: IGV views of stuctural variants identified in four specimens

Note: (A) Specimen with phenotype CcEe mf, carrying the *RHCE*Ce* and *RHCE*cE* alleles.

(B) Specimen with phenotype D--, carrying the *RHCE*CeN.08* and *RHCE*NEW* alleles.

(C) Specimen with phenotype D--, carrying the *RHCE*CeN.08* and *RHCE*CeN.07* alleles.

(D) Specimen with phenotype D--, carrying the *RHCE*NEW* and *RHCE*CeN.07* alleles.
